# Overexpression of miR-25 Downregulates the Expression of ROBO2 in Idiopathic Intellectual Disability

**DOI:** 10.3390/ijms25073953

**Published:** 2024-04-02

**Authors:** Rosa María Ordoñez-Razo, Yessica Gutierrez-López, María Antonieta Araujo-Solis, Gloria Benitez-King, Israel Ramírez-Sánchez, Gabriela Galicia

**Affiliations:** 1 Unidad de Investigación Médica en Genética Humana, Hospital de Pediatría “Dr. Silvestre Frenk Freund”, Centro Médico Nacional Siglo XXI, Instituto Mexicano del Seguro Social, Av. Cuauhtémoc 330, Col. Doctores, Mexico City CP 06725, Mexico; yessicagutierrezlopez09@gmail.com (Y.G.-L.); psic_ggr@hotmail.com (G.G.); 2 Departamento Clínico de Genética Médica, Hospital de Pediatría “Dr. Silvestre Frenk Freund”, Centro Médico Nacional Siglo XXI, Instituto Mexicano del Seguro Social, Av. Cuauhtémoc 330, Col. Doctores, Mexico City CP 06725, Mexico; tonyarasol@yahoo.com.mx; 3 Laboratorio de Neurofarmacología, Subdirección de Investigaciones Clínicas, Instituto Nacional de Psiquiatría “Ramón de la Fuente Muñiz”, Calzada México Xochimilco No. 101, Col. San Lorenzo Huipulco, Mexico City CP 14370, Mexico; bekin54@hotmail.com; 4 Sección de Estudios de Posgrado e Investigación, Escuela Superior de Medicina, Instituto Politécnico Nacional, Mexico City CP 07738, Mexico; israel.ramirez14@hotmail.com

**Keywords:** idiopathic intellectual disability, neural progenitor cells, olfactory neuroepithelium, miR-25, ROBO2

## Abstract

Idiopathic intellectual disability (**IID**) encompasses the cases of intellectual disability (ID) without a known cause and represents approximately 50% of all cases. Neural progenitor cells (NPCs) from the olfactory neuroepithelium (NEO) contain the same information as the cells found in the brain, but they are more accessible. Some miRNAs have been identified and associated with ID of known etiology. However, in idiopathic ID, the effect of miRNAs is poorly understood. The aim of this study was to determine the miRNAs regulating the expression of mRNAs that may be involved in development of **IID**. Expression profiles were obtained using NPC–NEO cells from IID patients and healthy controls by microarray. A total of 796 miRNAs and 28,869 mRNAs were analyzed. Several miRNAs were overexpressed in the **IID** patients compared to controls. miR-25 had the greatest expression. In silico analysis showed that ROBO2 was the target for miR-25, with the highest specificity and being the most down-regulated. In vitro assay showed an increase of miR-25 expression induced a decrease in ROBO2 expression. In neurodevelopment, ROBO2 plays a crucial role in episodic learning and memory, so its down-regulation, caused by miR-25, could have a fundamental role in the intellectual disability that, until now, has been considered idiopathic.

## 1. Introduction

Intellectual Disability (ID) is a neurodevelopmental disorder, characterized by significant limitations in intellectual functioning and adaptive behavior, present in 1–3% of the general population [1]. In 2013, the American Psychiatric Association developed a classification system for ID based on the level of intellectual impairment, which is why four categories have been described: mild ID refers to people with an IQ between 51 and 70; moderate ID for an IQ between 36 and 50; severe ID for an IQ between 20 and 35, and deep ID for an IQ below 20 [2].

In the last 15 years, individual genes have been identified as important factors in the etiology of ID, autism, and other pathologies related to abnormal neurodevelopment. However, despite these efforts, approximately 50% of IDs continue to be of unknown cause; therefore, these are known as idiopathic intellectual disabilities (**IIDs**) [3]. 

Alterations in processing, transporting, and regulating messenger RNAs (mRNAs) during neurodevelopment have been associated with the development of various pathologies, including ID. These changes can affect the growth and maturation of intraneuronal connections [4,5]. Therefore, the molecules involved in the regulation of these mRNAs may, in turn, represent an etiological factor, such as the case of the microRNAs (miRNAs), which regulate the expression of genes by binding to the UTR region of the target mRNA, causing the inhibition of the synthesis of proteins or the degradation of mRNAs [6]. The miRNAs are highly conserved; with 70% expressed in the brain [7] and an essential role in neurological development, neurotransmission, synaptic plasticity, and neurite outgrowth, they are indeed molecules that could represent a common etiological factor in cases of **IID**. Many studies have focused on analyzing the effect of miRNAs on the expression of mRNAs in syndromes related to abnormal neurodevelopment [8]. Some miRNAs have been identified in syndromes characterized by presenting intellectual disability or in ID of known etiology. For example, Williams syndrome is associated with miR-let-7f-2, miR-let-7g, miR-206, miR-125b, let-7c, and miR-200c [9], and Fragile X syndrome with miR-132, which was associated with the FMRP protein that affects neuronal morphology and synaptic strength [10]. miR-134, miR-25, and miR-137 have been directly related to the proliferation process of neural stem cells and neural progenitor cells [11,12,13]. Of these, miR-25 is part of the miR-106b-25 cluster found in the thirteenth intron of the gene encoding the Mcm7 protein, a member of a family of DNA helicases that is required for the replication process [13]. Overexpression of miR-25 has been shown to promote the proliferation of neural stem cells and neural progenitors in adult mice [13]. This effect may be due to direct regulation of the cell cycle inhibitor p57 [14]. Nevertheless, understanding of the effect of modifications in the expression of miRNAs in **IID** is poor, so it is essential to elucidate the etiological role of these in the development of **IID**.

Since studies in the brain of patients with **IID** are complicated due to tissue accessibility, the alternative is to use neural progenitor cells (NPCs) that can be obtained from the olfactory neuroepithelium (NEO), which is a neurogenic niche [15,16,17]. The NPCs are obtained from the NEO by exfoliation with a non-invasive procedure [18,19] and maintained in cell culture. In cell culture, these NPCs retain their characteristics of neural progenitor cells, as well as their ability to proliferate and the genetic and epigenetic information necessary to subsequently generate differentiated neural cells [18]. With the establishment of this methodology, it is now possible to study aspects of neurodevelopment, such as the regulation of mRNA expression by miRNAs in human pathologies, such as IID.

To elucidate which genes involved in human neurodevelopment undergo modified expression due to the overexpression of miRNAs in cases of **IID**, this work determined the expression profiles of miRNA and mRNA in NPCs derived from the NEO of patients with **IID** and healthy controls.

## 2. Results

### 2.1. Confirmation of the Neural Origin of Progenitors of the NPC Cell Cultures Derived from the NEO of **IID** Patients and Controls

**Figure 1:**
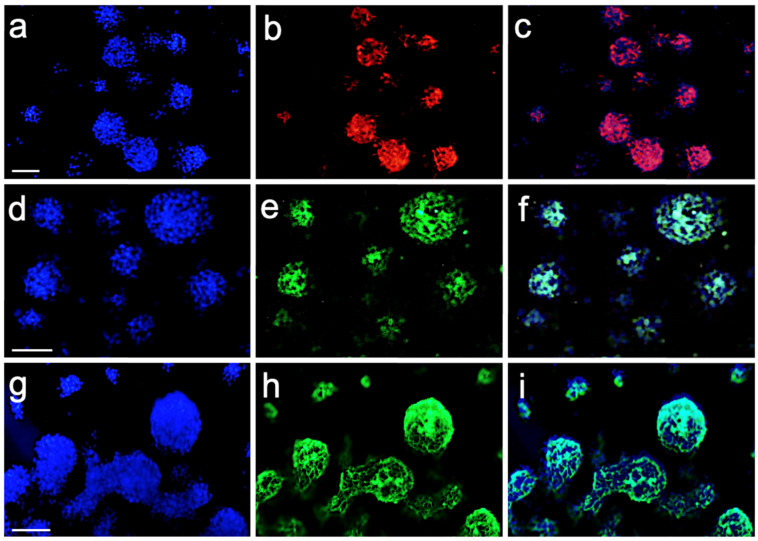
Neural-cell–marker expression in neurosphere cultures. Immunostaining of markers Sox2 with Cy3-red (**b**), Nestin with FITC-green (**e**), and βIII-tubulin with FITC-green (**h**). Nuclei were stained with DAPI (**a**,**d**,**g**). Merge DAPI/Sox2 (**c**), DAPI/Nestin (**f**) and DAPI/βIII-tubulin (**i**). Bar = 10 μm.

All cultures of olfactory neuroepithelium from 25 **IID** patients and 25 controls showed the formation of neurospheres. The neural origin of the progenitor from the obtained neurospheres was confirmed through expression analysis of the neural progenitor markers Sox2, Nestin, and βIII-tubulin (Figure 1). In the immunofluorescence assay, both Sox2 and Nestin markers (Figure 1b,e) showed similar expression in both patient and control cell cultures. This corroborates that the cultures contain progenitor cells of neural origin. The βIII-tubulin marker presented a lower signal (Figure 1h), indicating that the neurospheres in the cultures were principally undifferentiated cells.

### 2.2. Overexpression of miRNAs in NPCs of **IID** Patients

**Figure 2:**
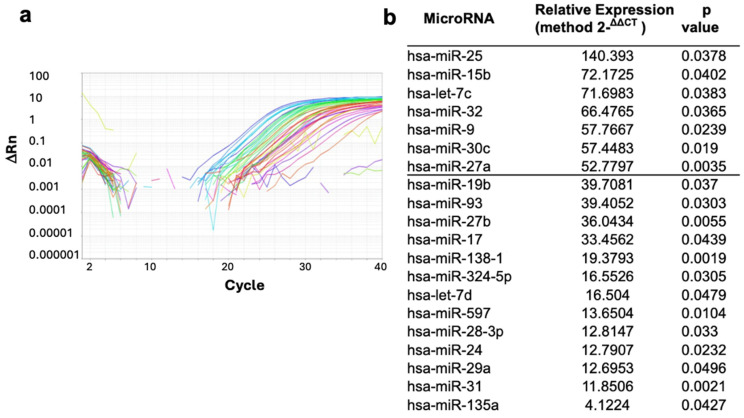
Expression analysis of the neurosphere miRNAs of **IID** patients and controls. (**a**) miRNA expression graph obtained for 754 miRNAs that were analyzed in each sample using TaqMan^®^ Array MicroRNA Cards (cards A and B). (**b**). List of the 20 miRNAs that were statistically significantly (*p* ≤ 0.05) overexpressed in IID patients compared to controls.

A total of 766 miRNAs were analyzed in all patients and controls. Only 245 miRNAs were expressed in both **IID** patients and controls (Figure 2a). The HTqPCR, gplots, and limma bioconductor statistical analyses showed that just 20 miRNAs were significantly different (*p* ≤ 0.05) in IID patients compared to controls (Figure 2b), which were miR-25, miR-15b, let-7c, miR-32, miR-9, miR-30c, miR-27a, miR-19b, miR-93, miR-27b, miR-17, miR-138-1*, miR-324-5p, let-7d, miR-597, miR-28-3p, miR-24, miR-29a, miR-31 and miR-135a*. Of these, miR-25, miR-15b, let-7c, miR-32, miR-9, miR-30c, and miR-27a showed levels of expression 50 times more in **IID** patients than controls (Figure 2b). miR-25 presented the highest expression; therefore, it was used to analyze its possible targets and effects on **IID**.

### 2.3. mRNA Expression Profile and Selection of Targets for miR-25

**Figure 3:**
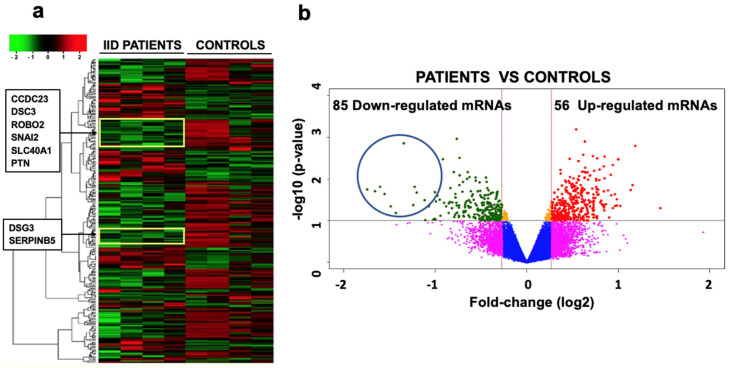
mRNAs expression profile from the neurospheres of four IID patients and four controls, representative of all samples analyzed. (**a**) The image shows the unsupervised hierarchical data pooling of 28,869 mRNAs for each sample. Colors represent expression levels of each mRNA; the red regions are the highly expressed genes (9829), the green regions are the weakly-expressed or nonexistent ones (11,361), and the black regions represent the genes without change (7679). (**b**) The volcano plot shows the expression of mRNAs from the 25 IID patients and 25 controls. In the scatter diagram, cut-off points were made in the values of fold-change (log2) at −0.26 and 0.26 to identify those genes that have changes in their expression that are valid by qRT-PCR and statistically significant (*p* ≤ 0.05 or 1 in −Log10). The most significant mRNAs are found at the top and appear symmetrically placed about the vertical line (Fch = 0). The 56 overexpressed genes are found in the upper-right corner and the 85 under-expressed genes appear in the upper-left corner. The yellow boxes in the heat map and the blue circle in the volcano plot show the location of some of the under-expressed mRNAs (including *ROBO2*) mentioned in this paper.

Of the 28,869 mRNAs that were analyzed, 21,190 showed changes in their expression when **IID** patients and controls were compared (Figure 3). In IID patients, 9829 were overexpressed (1.19 to 1.99 times higher) and 11,361 were under-expressed (−1.0 to −3.0 times lower) (Figure 3a). The statistical analysis of the mRNAs showed that only 85 mRNAs that were under-expressed and 56 that were overexpressed were significantly different (*p* ≤ 0.05; Figure 3b). 

In this work, we exclusively analyzed the under-expressed mRNAs found in all patients compared to the controls that have reported implications in neurodevelopment. 

In silico analysis with the DAVID database [20] provided the functional annotation, classification, and grouping of each of the 85 genes encoding mRNAs that were under-expressed. Of these 85 genes, those that have been reported to have an implication in neurodevelopment are *ROBO2*, *PTN*, *PAX7*, *TIMP1*, *ARHGEF5*, *MMP1*, *CDH13*, *SERPINB5*, *GPM6B*, *SNAI2*, *CTTNBP2*, *CCDC23*, *ERBB4*, *TP63*, *VSNL1*, *DSG3*, *SLC4A4*, *SEMA3E* (Table 1). 

The prediction of the target mRNAs of miR-25 was carried out by aligning the *ROBO2*, *PTN*, *PAX7*, *TIMP1*, *ARHGEF5*, *MMP1*, *CDH13*, *SERPINB5*, *GPM6B*, *SNAI2*, *CTTNBP2*, *CCDC23*, *ERBB4*, *TP63*, *VSNL1*, *DSG3*, *SLC4A4*, *SEMA3E* with the miR-25 sequences using the TargetScan [21], miRanda [22] and DIANA-microT 2023 [23] databases. Considering a score of mirSVR of −0.1 to −1.0, a value of 0.5–0.6 for the conserved sites (PhastCons), and the seed region of six to eight consecutive nucleotides, the best target mRNAs for miR-25 were *ROBO2*, which had the greatest statistical significance and the greatest number of alignments (in four regions: two with the seed sequence and another two regions with 12 and 15 nucleotides that included the complete seed); *ERBB4*, which aligned only once with 13 nucleotides; SLC4A4, which aligned in two positions with 6 and 11 nucleotides; SNAI2 and *TIMP1* genes which aligned at two positions with 10 and 16 nucleotides, respectively; and *CDH13*, *CTTNBP2*, *DSG3*, *TP63*, and *SEMA3E* mRNAs, which only aligned with the seed region (six nucleotides) of miR-25. *PTN*, *PAX7*, *ARHGEF5*, *MMP1*, *SERPINB5*, *GPM6B*, *CCDC23*, and *VSNL1* did not present alignments to the seed region of miR-25. Due to this data, it was determined that the main target of miR-25 was the mRNA of *ROBO2* (Table 2), which was therefore studied to show that it is negatively regulated by miR-25.

### 2.4. KEGG and GO Functional Annotation and Enrichment Analysis

**Figure 4:**
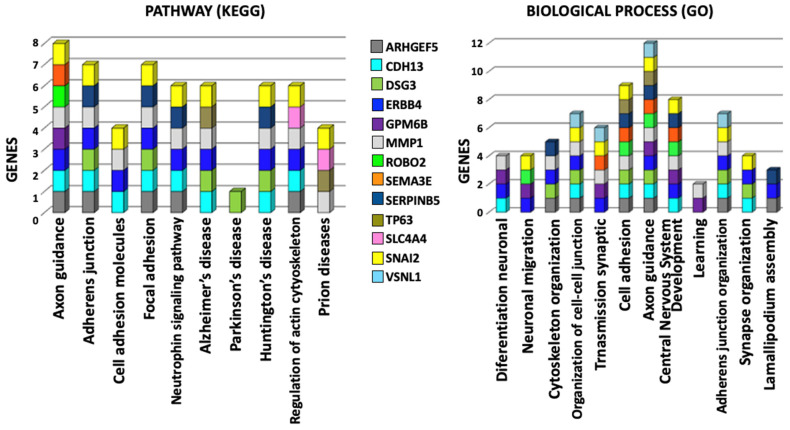
Analysis of pathways (KEGG) and biological processes (GO) of selected under-expressed genes. It was observed that axon guidance was the pathway and biological process in which most of the selected genes participate and, therefore, they all are associated with each other.

The Kyoto Encyclopedia of Genes and Genomes (KEGG) [24] and Gene Ontology (GO) [25,26] analyses of biological processes showed that these 13 gene targets for miR-25 are involved to an extent in axon guidance, adherent junctions, focal adhesion, development of the central nervous system, and synaptic transmission (Table 1 and Figure 4). The KEGG analysis showed that other cellular pathways are involved to a lesser extent, including those of adhesion molecules, focal adhesion, and neurotrophic signaling pathways, the regulation of actin in the cytoskeleton, and some associated diseases, such as Alzheimer’s, Huntington’s, and Prion disease; GO analysis showed biological processes such as neural differentiation, neural migration, cytoskeleton organization, cell–cell junction organization, etc. All of these are related and associated with each other. It should be noted that *ROBO2* plays a part in the axon guidance pathway, cell adhesion, and central nervous system development (Figure 4).

### 2.5. Validation of Expression Obtained in the Microarray of the ROBO2 mRNA by qRT-PCR

**Figure 5:**
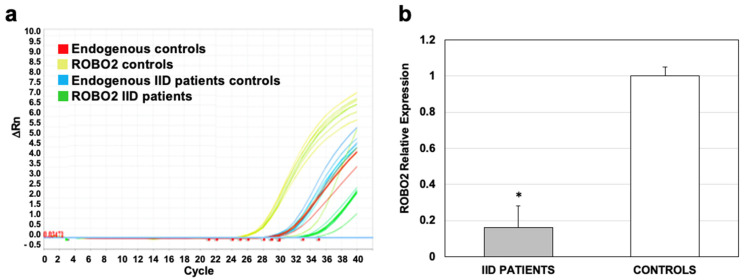
Validation of *ROBO2* expression in 25 **IID** patients and 25 controls by qPCR. (**a**) Graph of expression of *ROBO2* in patients and controls; green curves show the expression in patients and in yellow, the controls. (**b**) Relative expression of *ROBO2*; the expression value in controls was taken as the basal value, so the value of 1 was assigned to it. * *p* ≤ 0.05.

The *ROBO2* expression obtained from the microarray was validated by qRT-PCR in eight IID patients and eight controls, randomly selected. The amplification of mRNAs of **IID** patients and controls showed that the expression of *ROBO2* (green curves, Figure 5a) is much lower than in the controls (yellow curves, Figure 5a). When calculating the value of the expression of the patients compared to the controls, it was found that the expression in the IID patients is at least six times lower and the difference in the expression of ROBO2 was significant with *p* ≤ 0.05 (Figure 5b). The relative expression of the controls was taken from the baseline, so it was assigned a value of 1 (Figure 5b). This analysis corroborated the down-regulation of *ROBO2* in **IID** patients observed in the microarray.

### 2.6. Down-Regulated ROBO2 Expression by miR-25

**Figure 6:**
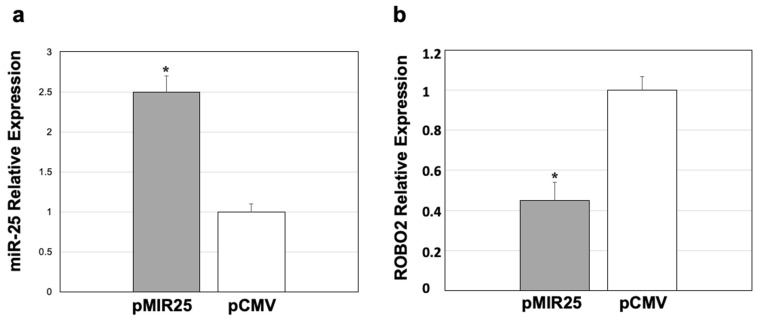
Relative expression of miR-25 and *ROBO2* in NCI-H2087 cells after transfection with CMV-MIR25 and pCMV. (**a**) Relative expression of miR-25. (**b**) Relative expression of *ROBO2*. The expression value in pCMV was taken as the baseline, so the value of 1 was assigned to it for both cases. * *p* ≤ 0.05.

To analyze the regulatory effect of miR-25 overexpression on *ROBO2* expression, an in vitro assay was performed on the NCI-H2087 (human lung carcinoma) cell line, given that these cells maintain constantly expressed *ROBO2* and miR-25 [27,28]. The transfection of the plasmid pMIR25 in the NCI-H2087 cells showed that the relative expression of miR-25 was 1.5 times higher (Figure 6a) compared to its expression when the cells were transfected with an empty vector (pCMV). For this analysis, the basal expression of miR-25 was considered with a value of 1 (Figure 6a). This demonstrated that the expression of miR-25 in NCI-H2087 cells transfected was augmented. At the same time, the expression of *ROBO2* was analyzed by qRT-PCR in these same transfected cells to determine if the expression of *ROBO2* could be regulated by the elevated expression of miR.25. This analysis showed that *ROBO2* expression levels were lower in cells with miR-25 overexpression (pMIR25-transfected) compared to cells that were transfected with pCMV (Figure 6b). The decrease was more than half of the basal *ROBO2* expression in these cells, which could be seen in the pCMV transfection (Figure 6b). These results suggest a correlation between miR-25 overexpression and down-regulation of *ROBO2* expression and that this regulation could be a crucial factor in **IID**.

## 3. Discussion

This report is the first to analyze the association of the overexpression of miRNAs with the down-regulation of genes that participate in critical stages of neurodevelopment and axon guidance in progenitor cells of neural origin derived from the olfactory neuroepithelium of patients with idiopathic intellectual disability. Also, it is the first to show that the overexpression of miR-25 induces the under-expression of the *ROBO2* mRNA, which can be crucial in neurodevelopment and axon guidance. Therefore, its down-regulation may play a transcendental role in **IID**.

The expression of *ROBO2* is fundamental since it is involved in axon guidance, neocortical development, and participates in the proliferation, migration, and maturation of cortical neurons during development [29]. *ROBO2* is crucial for establishing synaptic connectivity in the hippocampus, which is essential in episodic learning and memory [29]. In this context, a decrease in *ROBO2* expression can cause a deficit in neurological development, causing various disorders, such as intellectual disability, schizophrenia, epilepsy, learning disabilities, Parkinson’s disease, and dyslexia, as well as affecting other fundamental biological processes [30,31,32,33,34,35,36]. 

In patients with autism spectrum disorder (ASD), reduced levels of mRNA and proteins of axonal guidance signal receptors, including *PLXNA4* and *ROBO2*, have been found in the anterior cingulum cortex, which transmits neural signals between the cerebral hemispheres and has been related to social cognition [37], a characteristic also related to ID. 

*ROBO2* is a receptor for the Slit1 and Slit2 proteins, both of which have an essential role in axonal guidance, but it is also known that the absence of these proteins leads to a loss of mitosis in the ventricular zone. The *ROBO2*/Slit pathway was reported to have the ability to modulate progenitor cell dynamics in the developing brain and enhance the transition from primary to intermediate neural progenitor cells (IPCs) in the ventricular zone [38]. Therefore, the loss of *ROBO*/Slit signaling affects the development of the serotonergic and dopaminergic systems, which have already been implicated in the pathophysiology of ASD [38]. Moreover, the expression of *ROBO2* was reduced in patients diagnosed with ASD [39]; therefore, the under-expression of the *ROBO2* receptor could represent a sufficient factor to affect processes such as axonal guidance and neurogenesis. Additionally, it has been reported that *ROBO2* is an important participant in the evolution of the brain [40] and that it is also involved in the development of higher brain functions; so *ROBO2* turns out to be a key regulator in several points of the assembly and function of the neocortical circuit [30].

The *ROBO*/Slit signaling pathway is not exclusive to CNS development; it has also been proposed that it participates in the regulation and maturation of non-neuronal tissues and organs, such as the lung [41]. Additionally, their role as a tumor suppressor gene in lung and breast cancer is well-known [42,43]. In the lung carcinoma cell line NCI-H2087, miR-25 is overexpressed [28] and maintains slightly elevated levels of ROBO2 [27], which indicates that the overexpression of miR-25 must be above the values reported in NCI-H2087 to be able to down-regulate *ROBO2* expression. We demonstrated that when miR-25 is overexpressed more than 1.5 times in this cell line, *ROBO2* expression levels decrease by more than half, which shows that the miR-25 expression must be several times more to down-regulate the expression of *ROBO2*. This was observed in the IID patients, in whom the expression of miR-25 is extremely high compared to controls, so it can cause a decrease in *ROBO2*, which is normally overexpressed in the brain. The reduction of *ROBO2* expression by the overexpression of miR-25 in IID is of great importance given that *ROBO2* participates in the *ROBO*/Slit signaling pathway, which has fundamental roles in multiple events of neocortical development, from the proliferation of progenitor cells to circuit formation [44]. In addition, this alteration could be the central axis in IID and all the pathologies associated with these processes. 

In this context, we can suggest that the overexpression of miR-25 causes a decrease in *ROBO2* expression, which could affect essential processes during neurodevelopment and ultimately provoke **IID**. Although we are still far from identifying the specific etiological factors of **IID**, this report is undoubtedly the first step in proposing a possible participant in the etiopathogenesis of **IID**.

## 4. Materials and Methods

### 4.1. Study Subjects

Twenty-five samples of NEO by exfoliation were obtained from idiopathic intellectual disability (IID) patients and twenty-five healthy controls. Both patients and controls were school-aged children (6–15 years old). The patients’ ages were as follows: two were six years old, four were seven years old, two were eight years old, three were nine years old, six were ten years old, one was eleven years old, three were thirteen years old, two were fourteen years old, and two were fifteen years old. For the controls, the ages were equal to the patients because they were paired. To corroborate the *ROBO2* down-regulation observed in the IID patients’ samples, a microarray was carried out by qRT-PCR with TaqMan^®^ probes specific for the mRNA of *ROBO2* in eight patients and eight controls previously used in the microarray.

The participants were diagnosed according to the Diagnostic and Statistical Manual of Mental Disorders (DSM-IV), clinical data, and molecular tests. The mild intellectual disability patients were selected by the Wechsler Intelligence Scale with the intelligence quotient (IQ) equal to or less than 70; for the controls, the IQ was greater than 80. Patients and controls were referred from Departamento de Genética. Unidad Médica de Alta Especialidad, Hospital de Pediatría “Dr. Silvestre Frenk Freund” Centro Médico Nacional Siglo XXI, to the Unidad de Investigación Médica en Genética Humana in the same hospital, after informed consent acceptance. This study was approved by the Scientific and Ethics Committee of Health Research from the Instituto Mexicano del Seguro Social, Siglo XXI, with registration number R-2010-3603-33, and by the Declaration of Helsinki, 1964.

### 4.2. Sampling and Human Progenitor Neural Cell (NPC) Culture Characterization

The samples and NPC cultures (neurospheres) were obtained and characterized according to the methods reported by Jimenez-Vaca et al. [18]. The following primary antibodies were used for the characterization of the NPCs: anti-Nestin, anti-Sox2, anti-βIII-tubulin, and 4′,6′-diamidino-2-phenylindole dihydrochloride (DAPI) to stain the nuclei. All the antibodies used were manufactured by Zymed Invitrogen Corporation (Camarillo, CA, USA).

### 4.3. MicroRNA Expression Profile

MicroRNA was extracted from each patient and control sample neurospheres using the mirVana™ Kit (miRNA Isolation Kit, Invotrogen, Camarillo, CA, USA) according to the manufacturer’s instructions. The total miRNA extracted was quantified in a NanoDrop (Thermo Fisher Scientific, Waltham, MA, USA). To determine the expression profile of the miRNAs from patients and controls, a TaqMan^®^ Array Human MicroRNA Cards (cards A and B) specific to human miRNAs (Applied Biosystems, Waltham, MA, USA) was used. This assay allows accurate quantitation of 756 human microRNAs. Four endogenous controls (MammU6) on each card aid in data normalization. Also, Megaplex™ RT Primers, Human v2.0 (Applied Biosystems, Waltham, MA, USA), primers were used as necessary for the reaction. The procedure is based on an RT-PCR reaction, cDNA preamplification with TaqMan^®^ enzyme, and a dilution according to manufacturer.

### 4.4. mRNA Expression Profile

Total mRNA was obtained from 2.0 × 10^6^ neurospheres from each patient and each control using the Trizol technique. First, 1 mL of trizol was added to the cells, they were then lysed and incubated for 5 min at room temperature (RT). Next, 40 μL of chloroform was added, mixed vigorously for 15 s, and incubated for 5 min at RT. It was centrifuged at 12,000× rpm at 4 °C for 10 min. The aqueous phase was transferred to a new tube and 50 μL of isopropyl alcohol was added, mixed, and incubated at 4 °C for 10 min. It was centrifuged at 7500× rpm for 5 min and the pellet obtained was washed twice with 500 μL of 75% ethanol. Finally, the mRNA was resuspended in 30 μL of RNase-free water. The expression analysis was performed using the GeneChip^®^ Human Gene 1.0 ST Array (Affymetrix, Santa Clara, CA, USA). This involves an RT-PCR–based method and biotin labeling to obtain cRNA. The cRNA is fragmented for subsequent hybridization microarray control. An aliquot was taken to determine the size of the fragments, which should be 35 to 200 bases. For hybridization, labeled cRNA fragments are mixed with B2 oligonucleotide control (3 nM), 20× eukaryotic hybridization controls, 2× hybridization buffer, and DMSO, in a 300 μL final volume. The hybridization cocktail was heated to 99 °C for 5 min. Hybridization was maintained for 16 h and subsequently washed, stained, and scanned through the Fluidics Station 400 or 450/250 workstation. Data analysis was performed with DataAssist v1.0 software (the intensities for each probe in GeneChip Expression Analysis Fundamentals data).

### 4.5. Selection of Differentially-Expressed Genes with Function in the CNS and Neurodevelopment

The search for any relationship between genes with differential expression and some function in the CNS was carried out using the DAVID database [20], which allows annotation, classification, and functional grouping of each gene, in addition to determining the signaling and regulation pathways in which they are involved.

### 4.6. In Silico Prediction of Target Genes for miR-25

To identify which genes are targets for miR-25, an in silico alignment of each mRNA sequence with significant under-expression against the mature miR-25 was conducted. For the selection of a possible target of miR-25, the TargetScan [21], miRanda [22], and DIANA-microT 2023 [23] databases were used. The mRNAs that presented the best alignments in their 3’ UTR region with miR-25 were selected. 

### 4.7. Functional Annotation (KEGG) and GO Enrichment Analysis

The KEGG (Kyoto Encyclopedia of Genes and Genomes) [24] and the Gene Ontology (GO) [25,26] databases were used to analyze the under-expressed genes previously selected as targets of miR-25. These analyses allowed us to determine the pathways of molecular interactions as well as the processes, functions, and locations in which these genes participate within the cell.

### 4.8. Validation of ROBO2 Expression by qRT-PCR

To corroborate the expression data of the microarray of the selected target gene (*ROBO2*), which is the target best aligned to miR-25, validation was carried out by qRT-PCR with TaqMan^®^ probes specific for the mRNA of *ROBO2* in samples from 8 patients and 8 controls previously used in the microarray.

The cDNA synthesis was performed in two steps. In the first step, 2 ug of sample, 250 ng of random primers, 1 mL of 10 mM dNTPs mix, and RNase-free water were incubated at 65 °C for 5 min to obtain a total reaction volume of 12 mL after adding all the reagents. Subsequently, the solution was cooled on ice. In the second step, the previous solution was incubated at 37 °C for 2 min after adding 4 mL of 5× buffer, 2 mL of 0.1 M DTT, and 1 mL of RNaseOUT™ (Invitrogen, CA, USA) Recombinant Ribonuclease Inhibitor (40 units/mL). A total of 1μL of the enzyme M-MLV RT (200 units/mL Invitrogen, CA, USA) was added, and the solution was incubated at 25 °C for 10 min. It was then incubated at 37 °C for 50 min. Reverse transcription samples were stored at −20 °C until use. For real-time qPCR, the StepOne™ Real-Time System model from Applied Biosystems was used with the corresponding software and the expression assays from the same company, which are previously validated, optimized and exist for practically all the human genes. Reactions were carried out according to the manufacturer’s protocol. Each reaction (20 mL) contained 10 mL TaqMan Universal PCR Master Mix NoAmpErase^®^ UNG (Applied Biosystems, CA, USA) (2×), 1 mL TaqMan Gene Expression Assay Mix (20×), 8 mL RNase-free water, and 1 mL of sample ten-fold–diluted reverse transcription product. For each assay, a negative control was performed with RNase-free water, and the RPB1 subunit of RNA polymerase II (POLR2A) was used as an endogenous control. The quantification of the relative expression of the genes was carried out by the method of the second derivative 2^−ΔΔCt^. The TaqMan^®^ Gene Expression Assay IDs for *ROBO2* and internal controls were *ROBO2* (Hs00326067_m1) and POLR2A (Hs00172187_m1).

### 4.9. In Vitro Assay of miR-25 Overexpression and Its Effect on ROBO2 Expression

To determine the effect of miR-25 on *ROBO2* mRNA expression, miR-25 was overexpressed in the human lung carcinoma cell line NCI-H2087. The NCI-H2087 cells maintain both ROBO2 and miR-25 consistently expressed [27,28]. The overexpression of miR-25 in the NCI-H2087 cells was generated by the transfection of the pMIR25 plasmid, which was obtained by the insertion a sequence of 643 nucleotides that contained the pri-miR25 sequence (84 nucleotides) plus a sequence of 280 nucleotides on each flanking side (between the site Sgfl and Mlul) in the vector pCMV-MIR (Origene^®^, Rockville, MD, USA). As transfection control, empty pCMV-MIR (Origene^®^, MD, USA) was used.

(a) Culture of the Human Lung Carcinoma Cell Line NCI-H2087

The cell line NCI-H2087 (non-small cell lung cancer: adenocarcinoma, cat. CRL-5922), was acquired from the bank of the American Type Culture Collection (ATCC). The cell line was maintained in RPMI-1640 medium (ATCC, 30-2001) with 100 μg/mL penicillin/streptomycin (GIBCO 15140148), supplemented with 5% fetal bovine serum (FBS) (GIBCO 26140079), under a 5% CO_2_ atmosphere and a temperature of 37 °C. To ensure its continuity, periodic reseeding was carried out when reaching 70–90% cell confluence with a 0.05% trypsin/0.2% EDTA solution. A renewal of the culture medium was carried out 2 or 3 times per week.

(b) Transfection of pMIR25 and pCMV plasmids in NCI-H2087 Cells

Transfection was performed using Lipofectamine™ 2000 (Invitrogen). The assay was performed in 3 independent experiments, each with triplicates for each plasmid following the manufacturer’s recommended instructions.

(c) Relative Expression of miR-25 Transfected with the Plasmids

RNA from transfected cells was extracted 24 h after transfection by the Trizol method and cDNA specific for mature miR-25 was subsequently made with TaqMan^®^ MicroRNA Assay Protocol from Applied Biosystems, according to the manufacturer’s instructions. This system amplifies mature miRNAs in two steps: (1) miR-25 cDNA synthesis using specific primers in stem-loop and (2) quantification by real-time PCR using the chain elongation produced by the unfolding of the primer in stem-loop, which allows the binding of the 2 primers and 1 probe necessary for quantification. The quantification of the relative expression of miR-25 was carried out using the second derivative 2^−ΔΔCt^ method and a comparison of means was done using Student’s *t*-test for a statistical test (*p* ≤ 0.05).

(d) Relative Expression of *ROBO2* in the NCI-H2087 Cell Line Transfected with pMIR25 and pCMV

The RNA of the transfected cells was extracted by the Trizol method and, subsequently, the cDNA was made with the enzyme M-MLV RT using the protocol already described. Real-time PCR was performed with TaqMan^®^ probes using the StepOne™ Real-Time System model from Applied Biosystems with the corresponding software. The reactions were carried out according to the manufacturer’s protocol already described. The RPB1 subunit of RNA polymerase II (POLR2A) was used as an endogenous control. The quantification of the relative expression of the genes was carried out using the second derivative 2^−ΔΔCt^ method and the Student’s *t*-test to make a comparison of means as a statistical test (*p* ≤ 0.05).

## 5. Conclusions

In neurodevelopment, ROBO2 plays a key role in episodic learning and memory, so its probable down-regulation by the overexpression of miR-25 could have a crucial role in intellectual disability, which, until now, has been considered idiopathic. However, this suggestion must be corroborated with more studies.

## Figures and Tables

**Table 1:** Under-expressed mRNAs (−1.3- to −3.0-times less than the control ± standard deviation) in NPCs of 25 IID patients. These mRNAs were statistically significant (*p* ≤ 0.05) and have been implicated in neurodevelopment.

mRNA	Expression	*p* Value	Function
ROBO2	−1.50 ± 0.0294	0.006	Axon guidance and neurogenesis.
PTN	−1.60 ± 0.0937	0.008	Promotes neurites growth.
PAX7	−1.57 ± 0.1212	0.010	Neurogenesis.
TIMP1	−1.42 ± 0.0901	0.019	Formation of the cerebral cortex and memory.
ARHGEF5	−1.50 ± 0.0654	0.019	Associated with Rho protein (important in Alzheimer’s).
MMP1	−1.50 ± 0.0450	0.023	Projection of neurons and dendrites.
CDH13	−1.40 ± 0.0972	0.024	Neural projection/signaling receptor.
SERPINB5	−1.97 ± 0.6061	0.025	Adherent union in axon guide and dendrite formation.
GPM6B	−1.50 ± 0.0681	0.025	Neurogenesis.
SNAI2	−1.70 ± 0.2608	0.028	Cell adhesion and migration of the neural tube.
CTTNBP2	−1.39 ± 0.4588	0.028	Regulation of synaptic signaling.
CCDC23	−1.30 ± 0.5006	0.029	Synapse formation.
ERBB4	−1.37 ± 0.1877	0.029	Neural crest migration and axon guidance.
TP63	−1.93 ± 0.3308	0.031	Apoptosis in neurodevelopment.
VSNL1	−2.16 ± 0.1474	0.032	Neuronal morphology (Schizophrenia).
DSG3	−1.98 ± 0.2170	0.034	Junction adherents in the axon guide.
SLC4A4	−1.59 ± 0.1901	0.037	Synaptic vesicle formation.
SEMA3E	−1.59 ± 0.1877	0.042	Axon guide.

**Table 2:** Alignment of selected mRNAs with miR-25. The number of nucleotides and the number of sites of alignment for each mRNA that was under-expressed and related to neurodevelopment and the CNS of **IID** patients, as well as the values of mirSVR and PhastCons after alignment with the miR-25 sequence.

mRNA	Alignment	mirSVR	PhastCons
ROBO2	15nt, 12nt, 6nt (×2)	−0.9934	0.8038
PTN	-	-	-
PAX7	-	-	-
TIMP1	10nt, 6nt	−0.1734	0.6056
ARHGEF5	-	-	-
MMP1	-	-	-
CDH13	6nt	−0.0564	0.7202
SERPINB5	-	-	-
GPM6B	-	-	-
SNAI2	16nt, 10nt	−0.1044	0.6056
CTTNBP2	6nt		
CCDC23	-	-	-
ERBB4	16nt, 13nt	−0.0143	0.5669
TP63	6nt	−0.0580	0.6197
VSNL1	-	-	-
DSG3	12nt, 6nt		
SLC4A4	11nt, 6nt (×2)	−0.1022	0.5950
SEMA3E	6nt (×2)	−0.0233	0.5001

## Data Availability

The data presented in this study are available upon request from the corresponding author due to restrictions of privacy.
